# Supplementary material for: SyNDI: synchronous network data integration framework
Source: BMC Bioinformatics. 2018 Nov 6;19:403. doi: 10.1186/s12859-018-2426-5 (PMC6219086; doi:10.1186/s12859-018-2426-5)
Supplement: Supplementary file 5 — Files of the example presented in Additional file 4. This additional file is a zip package that contains all files used in Additional file 4. Detailed descriptions of each of these files are presented in a README file included in this zip package. (GZ 30411 kb) [file 12859_2018_2426_MOESM5_ESM.gz › SyNDITools/Meme2Fimo/meme_4.9.0_4.tar.gz!/meme_4.9.0/doc/fimo.xml]

FIMO - a motif search tool


**Usage:**

`fimo [options] <motifs> <database>`

**Description:**

The name `FIMO` stands for "Find Individual Motif Occurences."
The program searches a sequence database for occurrences of known motifs,
treating each motif independently.
The program uses a dynamic programming algorithm to convert log-odds scores (in bits) into p-values,
assuming a zero-order background model.
By default the program reports all motif occurrences with a p-value less than `1e-4`.
The threshold can be set using the `--thresh` option.
The p-values for each motif occurence are converted to q-values following the method of
Benjamini and Hochberg ("q-value" is defined as the minimal
false discovery rate at which a given motif occurrence is deemed significant).
The `--qv-thresh` option directs the program to use q-values rather than p-values
for the threshold.
If a motif has the `strand` feature set
to `+/-` (rather than `+`), then
`fimo` will search both strands for occurrences.

The parameter `--max-stored-scores` sets the maximum number of motif
occurrences that will be retained in memory.
It defaults to 100,000.
If the number of matches found reaches the maximum value allowed,
FIMO will discard 50% of the least significant matches,
and new matches falling below the significance level of the retained matches
will also be discarded.

`FIMO` can make use of position specific priors (PSP) to improve its
identification of true motif occurrences.
To take advantage of PSP in FIMO you use must provide two command line options.
The `--psp` option is used to set the name of a
MEME PSP file,
and the `--prior-dist` option is used to set the name of a file
containing the binned distribution of priors.

**Input:**

- `<motifs>` is the name of a file containing
  a list of motifs, in
  MEME format.
- `<database>` is the name of a file containing
  a collection of sequences in
  FASTA format.
  The character `-` can be used to indicate that the sequence data
  should be read from standard input.
  This can only be used if the motif file contains a single motif.

The FASTA header lines are used as the source of sequence names.
The sequence name is the string following the initial '>' up
to the first white space character.
If the sequence name is of the form: `text:number-number`,
the text portion will be used as the sequence name.
The numbers will be used as genomic coordinates,
and the first number will be used as the coordinate of the first position of the sequence.
In all other cases the coordinate of the first postion of the sequence is
take as 1.

**Output:**

FIMO will create a directory, named `fimo_out` by default.
Any existing output files in the directory will be overwritten.
The directory will contain:

- An XML file named `fimo.xml` using the
  CisML schema.
- An HTML file named `fimo.html`
- A plain text file named `fimo.text`
- A plain text file in
  GFF format
  named `fimo.gff`

The default output directory can be overridden using the `--o` or `--oc`
options which are described below.

The `--text` option will limit output to plain text sent to the standard output.
This will diable the calcualtion of q-values.

The score reported in the GFF output is `min(1000, -10*(log10(pvalue)))`.

The HTML and plain text output contain the following columns:

- The motif identifier
- The sequence identiifer
- The strand
  `+` indicates the motif matched the forward strand,
  `-` the reverse strand,
  and `.` indicates strand is not applicable (as for amino acid sequences).
- The start position of the motif occurence (closed, 1-based coordinates, unless genomic coordinates are provided)
- The end position of the motif occurence (closed, 1-based coordinates, unless genomic coordinates are provided).
- The score for the motif occurence.
  The score is computed by by summing the appropriate entries from each column of
  the position-dependent scoring matrix that represents the motif.
- The p-value of the motif occurence.
  The p-value is the
  probability of a random sequence of the same length as the motif
  matching that position of the sequence with a score at least as good.
- The q-value of the motif occurence.
  The q-value is the estimated
  false discovery rate if the occurrence is accepted as significant.
  See Storey JD, Tibshirani R. Statistical significance for genome-wide studies.
  *Proc. Natl Acad. Sci. USA (2003) 100:9440–9445*
- The sequence matched to the motif.

The HTML and plain text output is sorted by increasing p-value.

**Options:**
